# Dental Instruments

**Published:** 1867-05

**Authors:** 


### DENTAL INSTRUMENTS.

We recently had occasion to look through Dr. S. S. White's large and extensive stock of Dental goods at Philadelphia, and amongst other things that attracted our attention, was the arrangement and classification of excavators and pluggers which he had just completed. It consists in numbering all of any given class of instruments. The excavators, for instance, run from one to sixty, or perhaps more, embracing any shape and size, each having its fixed number. Every one will see at a glance the convenience of such an arrangement. Any one ordering these instruments, and knowing what he wants, can have his demands supplied as well as though he were present.

In the finish of these instruments there is quite an improvement in the bronzing of the handles, making a much neater instrument, besides obviating rust.

We would suggest that the profession aid Dr. White in carrying out his system, by becoming posted in it, and then shaping their orders accordingly. We presume that all Depots obtaining goods of him will adopt the same system. Dr. W. will soon issue the largest and most complete catalogue of dental goods ever published.

